# Epstein-Barr virus EBNA2 directs doxorubicin resistance of B cell lymphoma through CCL3 and CCL4-mediated activation of NF-κB and Btk

**DOI:** 10.18632/oncotarget.14243

**Published:** 2016-12-27

**Authors:** Joo Hyun Kim, Won Seog Kim, Jung Yong Hong, Kung Ju Ryu, Seok Jin Kim, Chaehwa Park

**Affiliations:** ^1^ Research Institute for Future Medicine, Samsung Medical Center, Sungkyunkwan University School of Medicine, Seoul 06351, Korea; ^2^ Division of Hematology and Oncology, Department of Medicine, Samsung Medical Center, Sungkyunkwan University School of Medicine, Seoul 06351, Korea; ^3^ Department of Oncology, Asan Medical Center, University of Ulsan College of Medicine, Seoul 05505, Korea; ^4^ Department of Health Science and Technology, Samsung Advanced Institute for Health Sciences and Technology, Sungkyunkwan University, Seoul 06351, Korea

**Keywords:** EBNA2, CCL3, CCL4, doxorubicin resistance, B cell lymphoma

## Abstract

Epstein-Barr virus (EBV)-encoded nuclear antigen, EBNA2, expressed in EBV-infected B lymphocytes is critical for lymphoblastoid cell growth. Microarray profiling and cytokine array screening revealed that EBNA2 is associated with upregulation of the chemokines CCL3 and CCL4 in lymphoma cells. Depletion or inactivation of CCL3 or CCL4 sensitized DLBCL cells to doxorubicin. Our results indicate that EBV influences cell survival via an autocrine mechanism whereby EBNA2 increases CCL3 and CCL4, which in turn activate the Btk and NF-κB pathways, contributing to doxorubicin resistance of B lymphoma cells. Western blot data further confirmed that CCL3 and CCL4 direct activation of Btk and NF-κB. Based on these findings, we propose that a pathway involving EBNA2/Btk/NF-κB/CCL3/CCL4 plays a key role in doxorubicin resistance, and therefore, inhibition of specific components of this pathway may sensitize lymphoma cells to doxorubicin. Evaluation of the relationship between CCL3 expression and EBV infection revealed high CCL3 levels in EBV-positive patients. Our data collectively suggest that doxorubicin treatment for EBNA2-positive DLBCL cells may be effectively complemented with a NF-κB or Btk inhibitor. Moreover, evaluation of the CCL3 and CCL4 levels may be helpful for selecting DLBCL patients likely to benefit from doxorubicin treatment in combination with the velcade or ibrutinib.

## INTRODUCTION

Epstein-Barr virus (EBV) preferentially infects B lymphocytes and transforms resting B cells into permanent, latently infected lymphoblastoid cell lines [1, 2, 3]. EBV comprises EBV nuclear antigen (EBNA 1, 2, 3A, 3B, 3C and –LP) and latent membrane (LMP 1, 2A and 2B) proteins. EBV-infected cells express a group of nuclear proteins that influence both viral and cellular transcription. Among these, EBNA2 plays an important role in B-cell transformation through transcriptional regulation of CD23 and the key viral genes, LMP1 and LMP2A [4, 5, 6].

Elevated levels of CCL3 and CCL4, also known as macrophage inflammatory protein-1α (MIP-1a) and -1β (MIP-1b) [7], have been detected in EBV-associated malignancies [8, 9, 10]. Both CCL3 and CCL4 are increased via BCR signaling and highlighted as biomarker genes in B cells [11, 12]. Moreover, elevated CCL3 and CCL4 plasma levels represent independent prognostic markers in chronic lymphocytic leukemia (CLL) patients [13]. CCL3 and CCL4 levels in patients with CLL are rapidly normalized after pharmacological inhibition of BCR signaling with idelalisib [14] or ibrutinib [15]. In diffuse large B cell lymphoma (DLBCL), CCL3 has been validated as one of the six most powerful independent predictors of survival [18]. CCL3 and CCL4 are associated with proliferation of B cells [19]. However, EBNA2 is yet to be explored as a therapeutic biomarker for drug treatment, despite evidence from earlier EBNA2 and BCR signaling-related studies.

In the present investigation, we addressed the functional consequences of EBNA2 expression in B cell lymphoma cell lines. Data from cDNA microarray and cytokine array analyses confirmed that EBNA2 increases CCL3 and CCL4 expression in DLBCL cells, in turn leading to increased survival signaling, including via the Btk and NF-κB pathways. Treatment with ibrutinib and velcade effectively prevented the upregulation of CCL3 and CCL4 induced by EBNA2, supporting the involvement of Btk and NF-κB signaling in cell survival. We further investigated the effects of doxorubicin alone and in combination with these inhibitors on DLBCL cells. The synergistic effects of the drug combinations further support the targeting of Btk or NF-κB as a promising strategy to enhance the therapeutic efficacy of drugs for EBV-positive DLBCL with high levels of circulating CCL3 or CCL4.

## RESULTS

### EBNA2 increases CCL3 and CCL4 expression in DLBCL cells

**Figure 1:**
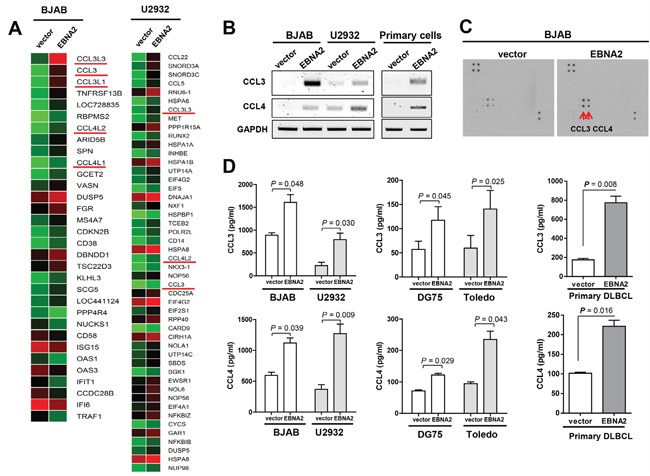
Induction of CCL3 and CCL4 expression by EBNA2 **A**. Gene expression analysis of EBNA2-containing BJAB and U2932 cells, compared to empty vector control cells. **B**. BJAB, U2932, and primary cells were transiently transfected with EBNA2 or empty vector for 24 h. RT-PCR was performed to determine CCL3 and CCL4 mRNA levels according to EBNA2 expression. **C**. EBNA2-induced upregulation of CCL3 and CCL4. Cytokine expression in BJAB cells expressing EBNA2 or control vector. Individual cytokines were spotted in duplicate, and the identities of CCL3 and CCL4 indicated. Positive control spots are located at the corners of the human cytokine array. **D**. ELISA analysis of CCL3 and CCL4 protein levels in culture supernatants. Data represent mean values ± SEM of three independent experiments. Each experiment was performed with triplicate samples.

EBNA2 is a 497 residue-long nuclear protein that acts as a transcriptional activator. To identify the genes increased by EBNA2, we performed genome-wide analysis of gene transcripts using Illumina Human HT-12 v4 Expression BeadChip. Notably, the expression levels of 22 genes, including CCL3 (CCL3L3, CCL3, CCL3L1, CCL4L2, CCL4L1), were augmented more than two-fold in EBNA2-expressing cells, BJAB and U2932 (Supplementary Table 1, 2), as presented in Figure 1A. EBNA2 enhanced CCL3 and CCL4 mRNA expression in EBNA2-expressing B cells to a significant extent, compared to control cells. We confirmed EBNA2-mediated induction of CCL3 and CCL4 in two EBV-negative lymphoma cell lines, BJAB and U2932 (Figure 1B). Cytokine array data additionally revealed specific upregulation of CCL3 and CCL4 proteins by EBNA2 in BJAB cells (Figure 1C). To confirm the EBNA2-induced increase in CCL3 and CCL4 proteins, the cytokine levels in culture medium were evaluated using ELISA. As shown in Figure 1D, large amounts of CCL3 and CCL4 accumulated in the culture medium of EBNA2-expressing cell lines (BJAB, U2932, DG75, and Toledo) and primary B cell lymphoma cells.

### Autocrine CCL3 and CCL4 play a critical role in EBNA2-induced survival of DLBCL cells

**Figure 2:**
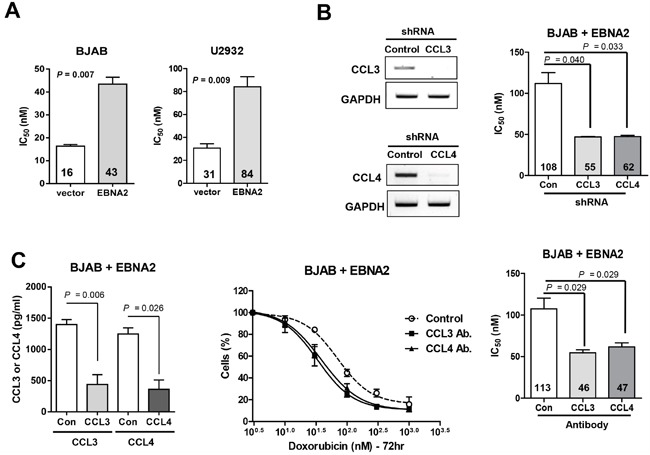
CCL3 and CCL4 contribute to doxorubicin resistance in DLBCL cells **A**. EBNA2 enhances cell survival following exposure to doxorubicin. BJAB and U2932 cells were transiently transfected with EBNA2-containing or empty vector, and treated with the indicated doses of doxorubicin for 48 h, followed by the trypan blue assay. **B**. CCL3 and CCL4 knockdown sensitizes EBNA2-expressing DLBCL cells to doxorubicin. BJAB cells were transiently transfected with EBNA2. After 24 h incubation, EBNA2-expressing cells were infected with shRNA, treated with doxorubicin for 72 h, and subjected to the CCK-8 assay. RT-PCR was performed to confirm shRNA efficacy. **C**. Blockade of CCL3 and CCL4 decreases survival of cells exposed to doxorubicin. BJAB cells were transiently transfected with EBNA2. After 24 h, EBNA2-expressing cells were seeded at a density of 3×10^5^ cells/well in 24-well plates and treated with 2.5 μg/ml CCL3 and CCL4 neutralization antibodies or control rabbit Ig. After 24 h incubation, cells were treated with doxorubicin for 72 h, followed by the CCK-8 assay. CCL3 and CCL4 protein concentrations in the culture supernatants of BJAB cells were determined using ELISA to confirm the efficacy of neutralization antibodies. Data represent mean values ± SEM of three independent experiments. Each experiment was performed with triplicate samples.

To determine the biological significance of our findings, we examined the effects of doxorubicin on survival of BJAB and U2932 cells overexpressing EBNA2. Within the BJAB cell group, the median inhibitory concentration (IC_50_) of doxorubicin was 43 nM in EBNA2-expressing cells whereas that for empty vector-containing control cells was 16 nM (*P* = 0.007; Figure 2A). Similar results were obtained with the U2932 cell line. The data indicate that EBNA2-overexpressing cells are less sensitive to doxorubicin than non-expressing cells transfected with control vectors. In view of the finding that EBNA2 enhances the survival of doxorubicin-challenged cells, we further examined whether CCL3 and CCL4 depletion can sensitize cells to the drug. EBNA2-expressing BJAB cells were transduced with lentiviral vectors to silence CCL3 and CCL4, and gene expression verified via RT-PCR (Figure 2B). As shown in Figure 2B, CCL3 and CCL4 mRNA levels were significantly decreased in knockdown cells. Silencing of CCL3 and CCL4 caused a dramatic reduction in EBNA2-mediated survival of cells after doxorubicin treatment for 72 h (IC_50_=55 nM and 62 nM, respectively), compared to control shRNA-transfected cells (IC_50_=108 nM). Similar results were obtained in the experiment with CCL3 and CCL4-neutralizing antibodies. BJAB cells were incubated with neutralizing antibodies to inhibit CCL3 and CCL4, and depletion of individual cytokines verified using ELISA (Figure 2C). Treatment with CCL3 or CCL4-neutralizing antibodies sensitized EBNA2-expressing BJAB cells to doxorubicin (P < 0.03; Figure 2C). These findings suggest that expression of EBNA2 enhances the resistance of cancer cells to doxorubicin, which may be at least partially dependent on upregulation of CCL3 and CCL4.

### Activation of Btk and NF-κB induces CCL3 and CCL4, which re-enhance phosphorylation of Btk and NF-κB

**Figure 3:**
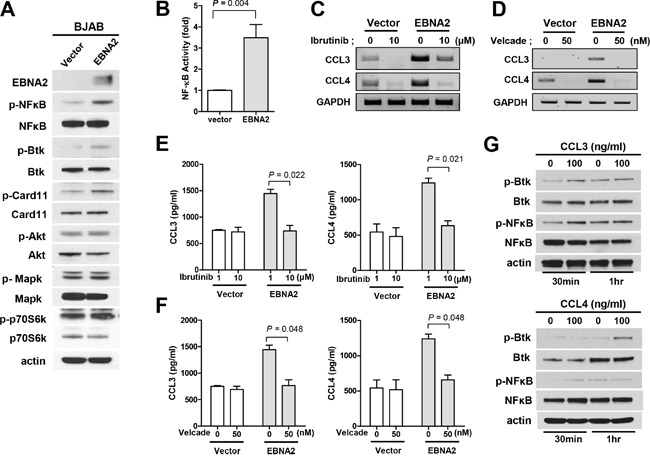
Association of CCL3/CCL4 expression with activation of Btk/NF-κB **A**. Western blot analysis of Btk, Card11, NF-κB, Akt, p44/42 Mapk and p70S6K phosphorylation according to EBNA2 expression. BJAB cells were transiently transfected with EBNA2 or empty vector. **B**. EBNA2 enhances NF-κB activity. BJAB cells were transiently transfected with plasmids encoding EBNA2 or empty vector. Individual samples were incubated for 24 h, and luciferase activities monitored to quantify NF-κB promoter activity. **C, E**. Ibrutinib, a Btk inhibitor, prevents EBNA2-induced upregulation of CCL3 and CCL4 expression. BJAB cells were transiently transfected with EBNA2 or empty vector and treated with ibrutinib (10 μM) for 24 h, followed by RT-PCR (C) and ELISA (E). **D, F**. Inhibition of NF-κB abrogates EBNA2-induced upregulation of CCL3 and CCL4 expression. BJAB cells were transiently transfected with EBNA2-containing or empty vector, and treated with velcade (50 nM) for 24 h, followed by RT-PCR (D) and ELISA (F). Data represent mean values ± SEM of three independent experiments. **G**. BJAB cells were treated with CCL3 and CCL4 human recombinant protein (100 ng/ml) in 1% FBS-containing RPMI for 30 min and 1 h. Activation of signaling proteins, including Btk and NF-κB, was observed using western blot.

CCL3 and CCL4 are known biomarkers for B cell receptor pathway activation [11, 12]. To explore the potential signaling pathways involved in EBNA2 activity, we examined the phosphorylation of signaling proteins, including Btk, Akt, Mapk, NF-κB, and mTOR. As shown in Figure 3A, EBNA2 promoted the phosphorylation of NF-κB and Btk. To address the requirement for Btk and NF-κB in EBNA2-mediated induction of CCL3 and CCL4, Btk and NF-κB inhibitors were employed. We analyzed the promoter activity of NF-κB according to EBNA2 expression to determine whether EBNA2 increases NF-κB promoter activity. As shown in Figure 3B, NF-κB activity was enhanced by EBNA2. Inhibition of Btk (ibrutinib) and NF-κB (velcade) efficiently blocked EBNA2-induced upregulation of CCL3 and CCL4 transcripts (Figure 3C and 3D). In addition, CCL3 and CCL4 production were decreased in the presence of velcade and ibrutinib in EBNA2-expressing, but not EBNA2-negative control BJAB cells (Figure 3E and 3F). To examine whether CCL3 and CCL4 enhance p-Btk and p-NF-κB, we used recombinant human CCL3 and CCL4 proteins. Treatment of BJAB cells with CCL3 and CCL4 for 30 min and 1 h resulted in Btk and NF-κB phosphorylation, as shown in Figure 3G.

### Inhibition of Btk or NF-κB sensitizes DLBCL cells to doxorubicin

**Figure 4:**
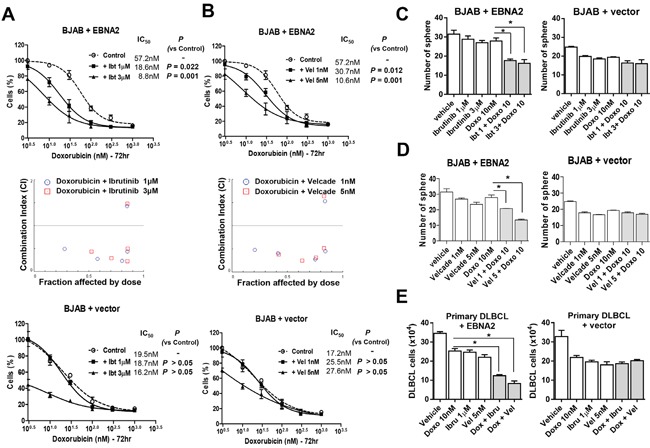
Inhibition of Btk or NF-κB sensitizes lymphoma cells to doxorubicin **A, B**. BJAB cells were transiently transfected with EBNA2-containing or empty vector and treated with the indicated doses of doxorubicin in the presence or absence of ibrutinib (72 h) or velcade (72 h). Cell viability was evaluated using the CCK-8 assay. The Combination Index (CI) value was calculated using CalcuSyn software (Biosoft, Ferguson, MO, USA). Antagonism is indicated by CI values greater than 1.1, additivity by values between 0.9 and 1.1, and synergism by values below 0.9. **C, D**. BJAB cells were transiently transfected with EBNA2-containing or empty vector and were treated with different concentrations of doxorubicin (10 nM) in the presence or absence of ibrutinib (1 and 3 μM) and velcade (1 and 5 nM). The sphere-forming assay was performed by seeding cells in 96-well plates and further observing for 2 weeks. *, *P* < 0.05 **E**. Primary ABC DLBCL cells were transiently transfected with EBNA2-containing or empty vector, and treated with the indicated doses of doxorubicin and inhibitors for 24 h. Cell viability was evaluated using the trypan blue assay. Data represent mean values ± SEM of three independent experiments. Each experiment was performed with triplicate samples. *, *P* < 0.05.

Notably, co-administration of doxorubicin and ibrutinib induced a significant decrease in the viability of EBNA2-expressing cells, but not control cells transfected with empty vector (Figure 4A). Similarly, growth inhibition was significantly enhanced upon combination with velcade in EBNA2-expressing cells, but not in control cells with empty vectors (Figure 4B). Moreover, combined treatment with doxorubicin and ibrutinib or velcade resulted in a highly synergistic reduction in cell viability with CI < 1.0. A Fraction-affected-Combination index (Fa-CI) plot was created using CompuSyn software. Consistently, co-treatment with doxorubicin and ibrutinib or velcade induced a marked decrease in sphere formation of EBNA2-expressing BJAB but not control cells transfected with empty vector (Figure 4C, 4D). Additionally, treatment of isolated primary cells from a DLBCL patient with a combination of doxorubicin and inhibitors (velcade or ibrutinib) was more efficacious, compared to doxorubicin or inhibitor alone (Figure 4E).

**Figure 5:**
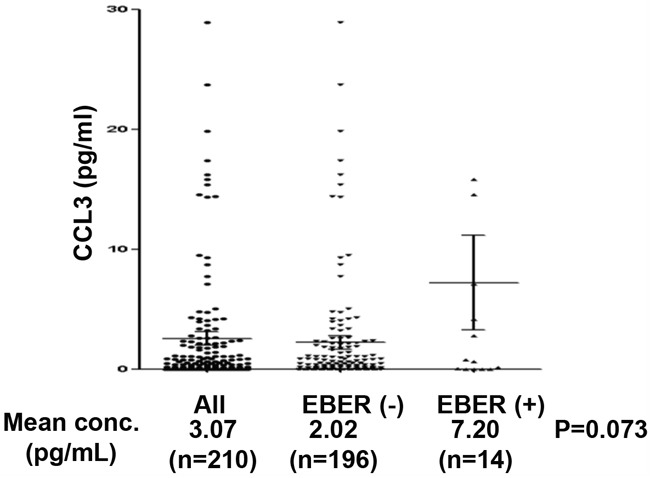
Dot plots of serum CCL3 levels according to EBER status Serum levels of CCL3 in all patients (n=210), those with EBER negativity (n=14, 6.7%), and those with EBER positivity (n=196, 93.3%).

Among a total of 210 patients with DLBCL, 14 (6.7%) showed EBER positivity and 196 (93.3%) showed EBER negativity. We observed a trend of higher mean serum CCL3 in EBER-positive compared with EBER-negative patients, as shown in Table 1 and Figure 5 (2.02 pg/mL versus 7.20 pg/mL, P=0.073). The data signify a close association of EBNA2-induced CCL3 and CCL4 upregulation with Btk and NF-κB activation pathways, supporting the potential utility of Btk and/or NF-κB as promising targets to overcome chemoresistance of EBV-positive DLBCL.

**Table 1:** Characteristics of the study samples (n = 210)

	All patients(n=210)		EBER (−)(n=196)		EBER (+)(n=14)	
**Age, no. (%)**						
60 y or less	123	(58.6)	119	(60.7)	4	(28.6)
Older than 60 y	87	(41.4)	77	(39.3)	10	(71.4)
**Sex, no. (%)**						
Male	128	(61.0)	122	(62.2)	6	(42.9)
Female	82	(39.0)	74	(37.8)	8	(57.1)
**Performance status, no. (%)**						
ECOG 0-1	173	(82.4)	162	(82.7)	11	(78.6)
ECOG 2-4	37	(17.6)	34	(17.3)	3	(21.4)
**Ann Arbor stage, no. (%)**						
Limited, I-II	103	(49.0)	97	(49.5)	6	(42.9)
Advanced, III-IV	107	(51.0)	99	(50.5)	8	(57.1)
**IPI risk group, no. (%)**						
Low/Low intermediate	130	(61.9)	124	(63.3)	6	(42.9)
High intermediate/High	80	(38.1)	72	(36.7)	8	(57.1)
**Bone marrow involvement**						
Negative	191	(91.0)	179	(91.3)	12	(85.7)
Positive	19	(9.0)	17	(8.7)	2	(14.3)
**Response to front-lineTx**						
CR or PR	181	(86.2)	171	(87.2)	10	(71.4)
SD or PD	29	(13.8)	25	(12.8)	4	(28.6)

ECOG; Eastern Cooperative Oncology Group, IPI; International Prognostic Index, CR; complete response, PR; partial response, SD; stable disease, PD; progressive disease.

## DISCUSSION

Epstein-Barr virus nuclear antigen 2 (EBNA2) is essential for the capacity of the virus to transform B cells, and mainly characterized as a transcription factor. EBNA2 transcriptionally activate cellular genes, such as CD23, and the key viral genes, LMP1 and LMP2A. Moreover, EBNA2 has been shown to contribute to EBV-induced B-cell transformation by altering miR expression [20] and cellular morphology, and increase colony formation in soft agar and tumorigenicity in nude mice [21]. In the present study, we identified the molecular targets of EBNA2 and examined the effects of specific EBNA2 targets on the drug response of EBV-infected B lymphoma cells. CCL3 and CCL4, known biomarkers for BCR pathway activation and prognosis in DLBCL, were selected using gene expression analysis. Based on DAVID software, a total of 20 GO functions were enriched. Among these, increased were mainly enriched in 10 GO functions (Supplementary Table 3), the most significant being regulation of immune response. KEGG pathway enrichment analysis revealed that increased DEGs were mainly enriched in two pathways, specifically, the chemokine signaling pathway and cytokine receptor interactions (Supplementary Table 4).

CCL3 and CCL4 expression has been detected in various B cell-related tumors, including multiple myeloma and chronic lymphocytic leukemia (CLL) [11, 22]. CCL3 enhances migration and signaling pathways mediating survival and proliferation in multiple myeloma cells [22, 23]. Recently, the *in vivo* importance of CCL3 and CCL4 expression in DLBCL for B cell receptor pathway activation and prognostic serum markers was highlighted [11, 12]. Chronic lymphocytic leukemia (CLL) patients contain elevated plasma CCL3 and CCL4 levels [13]. Btk, a component of the BCR pathway, is considered a potential novel target for the treatment of B cell lymphoma, and the Btk inhibitor, ibrutinib, has been successfully applied in clinical trials. Moreover, elevated CCL3 and CCL4 levels have been shown to be normalized by the Btk inhibitor in patients with CLL [14, 15], indicating that the BCR signaling pathway is responsible for chemokine production.

The transcription factor, EBNA2, clearly contributes to EBV-induced B-cell transformation and tumorigenicity [20, 21]. However, the precise relationship between EBNA2 and chemoresistance remains poorly understood at present. We showed here that EBNA2 increases doxorubicin resistance through enhancing activation of Btk and NF-κB and upregulating CCL3 and CCL4. Autocrine CCL3 and CCL4 induced by the oncoprotein LMP1 promote EBV-triggered B cell proliferation [19]. *In vivo*, EBNA2 also functions as a transcription factor to promote LMP1 expression [5]. Upon ectopic expression of EBNA2 alone, cells showed elevated CCL3 and CCL4 expression and doxorubicin resistance in our experiments. We found that overexpression of EBNA2 and its targets, CCL3 and CCL4, led to doxorubicin resistance, independent of the pathway underlying LMP1-mediated protection of cancer cells against chemotherapeutic agents [24–28]. Interestingly, CCL3 and CCL4 enhanced phosphorylation of Btk and NF-κB in DLBCL cells in culture, suggesting that upregulation of these chemokines may be the underlying cause of doxorubicin resistance.

Experiments from the current study showed that CCL3 and CCL4 are increased and responsible for Btk and NF-κB activation, and ultimately, chemoresistance of EBNA2-expressing DLBCL cells. Knockdown of CCL3 or CCL4 sensitized EBNA2-expressing cells to doxorubicin, further supporting a role in chemoresistance. In addition, selective inhibition of Btk or NF-κB effectively enhanced doxorubicin sensitivity, accompanied by downregulation of CCL3 and CCL4. Earlier reports have reported an association of high-dose doxorubicin with fatal congestive heart failure [29]. Combination treatment with ibrutinib or velcade may thus help to lower the treatment dose of doxorubicin required. While Ponader and co-workers have reported that ibrutinib decreases secretion of CCL3 and CCL4 in CLL cells [15], the cancer cell-autonomous survival function of these chemokines is yet to be explored. EBNA2 has been shown to promote NF-κB signaling [5, 30], which may, in turn, transactivate EBNA2 expression [4]. Accordingly, we suggest that EBV-positive DLBCL cells require Btk or NF-κB signaling to maintain survival via EBNA2-mediated upregulation of CCL3 and CCL4. EBV-positive DLBCL is more common in Asian (8–11%) than Western populations (<5%). Asian patients with EBV-positive DLBCL have unique and unfavorable clinical features, including older age, more advanced stage and higher International Prognostic Index (IPI), compared to patients with EBV-negative DLBCL. Moreover, survival of patients with EBV-positive DLBCL is significantly worse than that of the EBV-negative DLBCL group [31–33]. Our data provide mechanistic insights into the clinical activities of inhibitors targeting Btk and/or NF-κB in combination with doxorubicin for treatment of EBV-positive DLBCL, and support the utility of circulating CCL3 or CCL4 as response predictors to combination treatments in DLBCL.

## MATERIALS AND METHODS

### Cell lines, culture conditions, transfection and inhibitors

The U2932 cell line was purchased from the Leibniz-Institut DSMZ-Deutsche Sammlung von Mikroorganismen und Zellkulturen GmbH (Braunschweig, Germany). BJAB cells and expression plasmids encoding wild-type EBNA2 were obtained from Dr. M. S. Kang (Sungkyunkwan University, Korea). DG75 and Toledo cells were obtained from Dr. S. J. Kim (Sungkyunkwan University, Korea). Cell lines were cultured in RPMI 1640 supplemented with heat-inactivated 10% fetal bovine serum (FBS), penicillin and streptomycin (Gibco-BRL, Grand Island, NY) in a 5% CO_2_-containing atmosphere. Primary human ABC DLBCL cells were obtained from pleural effusion of a patient with primary ABC DLBCL using a protocol that the Samsung Medical Center Institutional Review Board approved, in accordance with the Declaration of Helsinki. CCL3 and CCL4 shRNA were purchased from Sigma-Aldrich (Milwaukee, WI), and CCL3 and CCL4-neutralizing Ab and recombinant human proteins from R&D systems (Minneapolis, MN). Lymphoma cell lines were transiently transfected with the EBNA2-expressing plasmid pSG5 using the Amaxa electroporation system (Amaxa, Gaithersburg, MD). Primary ABC DLBCL cells were transfected using Amaxa program X-01 with T solution. Ibrutinib was acquired from Selleck Chemicals (Houston, TX77054).

### cDNA microarray analysis

Genes expressed in EBNA2-containing and control BJAB and U2932 cells were analyzed on an Illumina HumanHT-12 v4.0 Expression BeadChip (Illumina, Inc., San Diego, CA). Target preparation and microarray processing procedures were performed as described in the Illumina Expression Beadchip Analysis Manual. The pre-processing procedure for cell intensity files (CEL) and subsequent microarray analyses were performed using Illumina GenomeStudio v2011.1 (Gene Expression Module v1.9.0). Data were subjected to global scale normalization. Differentially expressed genes were selected based on fold change and Student's *t*-test data (over two-fold and *P* <0.01, respectively), compared with the corresponding controls.

### Reverse transcriptase-polymerase chain reaction

Total RNA was prepared using a Qiagen RNA extraction kit (Qiagen, Valencia, CA), according to the manufacturer's instructions. For reverse transcription, 2 μg RNA was treated with RNase-free DNase, and cDNA obtained using an Omniscript RT kit (Qiagen). The cDNA generated was amplified using primers specific for CCL3 and CCL4. Glyceraldehyde 3-phosphate dehydrogenase (GAPDH) was amplified as the control. PCR products were visualized using 1.2% agarose gel electrophoresis in 1XTAE buffer containing SYBR Safe DNA Gel Stain (Invitrogen, Carlsbad, CA).

### Cytokine antibody array

Supernatant fractions of EBNA2- or vector-transfected B cells were collected after 24 h transfection, and applied to RayBio human cytokine antibody array 7 (RayBiotech, Inc., Norcross, GA) according to the manufacturer's instructions.

### ELISA

CCL3 and CCL4 protein concentrations in the culture supernatants were determined using an enzyme-linked immunosorbent assay (ELISA) kit (R&D Systems, Inc., Minneapolis, MN) in keeping with the manufacturer's instructions.

### NF-kB promoter-luciferase reporter assay

pGL3-NF-kB-Luc containing the luciferase gene under control of a NF-kB responsive element was obtained from Clontech (Mountain View, CA). The reporter construct and constitutively active Renilla reniformis luciferase-producing vector, pRL-TK (Promega, Madison, WI), were transfected into EBNA2-expressing BJAB cells via Amaxa electroporation. Firefly and Renilla luciferase activities were measured with the Dual-Luciferase Reporter Assay system, based on the manufacturer's instructions (Promega, Madison, WI).

### Assessment of cell viability

Drug effects on cell viability were monitored using trypan blue staining. Alternatively, the CCK-8 viability assay was performed. For the CCK-8 assay, cells were incubated in triplicate on a 96-well plate in the presence or absence of the indicated test samples within a final volume of 0.1 mL for 24 h at 37°C. Thereafter, 20 μL Cell Counting Kit-8 reagent (CCK-8; Dojindo Laboratories, Kumamoto, Japan) was added to each well. After 2 h incubation at 37°C, optical density (OD) at 450 nm was measured using a 96-well multiscanner autoreader. Cell viability was expressed as a percentage: (OD_[450 nm]_ of experimental sample/OD_[450 nm]_ of control).

### Antibodies for western blot

The antibodies employed for western blotting included those specific for p-p65(S536), p-Btk(S180), p-AKT(S473), AKT, p-CARD11(S652), p-p44/42 MAPK(T202/Y204), p44/42 MAPK, p-p70S6K(Thr389), p70S6K, (Cell Signaling, Beverly, MA) and EBNA2 (Millipore, Merrill JA).

### Sphere forming assay

The sphere formation assay was performed by seeding cells (100 cells/96-well plate) in BMSC supernatant. Cells were incubated in triplicate in the presence or absence of the indicated test samples in a final volume of 0.1 mL at 37°C. On day 14, spheres on the plate were counted under phase-contrast microscopy.

### Measurement of CCL3 levels in patients

The serum CCL3 concentration was measured in archived frozen samples of the Samsung Medical Center prospective cohort study (NCT#00822731). Archived serum sample aliquots stored at −80°C were thawed for use in the cytokine assay. The CCL3 concentration was measured in serum using the Procarta cytokine profiling kit (Panomics, San Diego, CA, USA), and all measurements performed in duplicate.

EBER was detected using an EBV ISH kit (Leica Microsystems, Bannockburn, IL, USA). We employed EBV-negative lymphoid tissues and the hybridization mixture without EBV oligonucleotides as the negative control. A positive reaction was defined as >20% cells showing nuclear positivity.

### Statistical analyses

All experiments were repeated three times unless otherwise stated. Data presented in graphs represent mean ± standard error of means from three independent experiments performed in triplicate. Differences between two mean values were analyzed using t-test and graphs produced using GraphPad Prism 6.

## SUPPLEMENTARY TABLES




