# Supplementary material for: Epstein-Barr virus EBNA2 directs doxorubicin resistance of B cell lymphoma through CCL3 and CCL4-mediated activation of NF-κB and Btk
Source: Oncotarget. 2016 Dec 27;8(3):5361–70. doi: 10.18632/oncotarget.14243 (PMC5354914; doi:10.18632/oncotarget.14243)
Supplement: Supplementary file 1 [file oncotarget-08-5361-s001.pdf]

Epstein-Barr virus EBNA2 directs doxorubicin resistance of B cell lymphoma through CCL3 and CCL4-mediated activation of NF-κB and Btk

SUPPLEMENTARY TABLES

Supplementary Table 1: List of upregulated genes by EBNA2 in BJAB cells

Gene symbol	Gene name	Fold change	Gene symbol	Gene name	Fold change
**CCL3L3**	chemokine (C-C motif) ligand 3-like 3	16.4	**VASN**	Vasorin	2.3
**CCL3**	chemokine (C-C motif) ligand 3	10.9	**DUSP5**	dual specificity phosphatase 5	2.3
**CCL3L1**	chemokine (C-C motif) ligand 3-like 1	10.4	**FGR**	FGR proto-oncogene, Src family tyrosine kinase	2.2
**TNFRSF13B**	tumor necrosis factor receptor superfamily, member 13B	5	**MS4A7**	membrane-spanning 4-domains, subfamily A, member 7	2.2
**LOC728835**	similar to cytokine, transcript variant 3	3.8	**CDKN2B**	cyclin-dependent kinase inhibitor 2B	2.2
**RBPMS2**	RNA binding protein with multiple splicing 2	3.3	**CD38**	CD38 molecule	2.2
**CCL4L2**	chemokine (C-C motif) ligand 4-like 1	3.2	**DBNDD1**	dysbindin domain containing 1	2.1
**ARID5B**	AT rich interactive domain 5B (MRF1-like)	2.6	**TSC22D3**	TSC22 domain family, member 3	2.1
**SPN**	Sialophorin	2.4	**KLHL3**	kelch-like family member 3	2.1
**CCL4L1**	chemokine (C-C motif) ligand 4-like 1	2.4	**SCG5**	secretogranin V	2.1
**GCET2**	germinal center-associated, signaling and motility	2.4	**LOC441124**	hypothetical LOC441124	2.0

Supplementary Table 2: List of upregulated genes by EBNA2 in U2932 cells

See Supplementary File 1

Supplementary Table 3: The GO functional enrichment analysis of DEGs in the threshold of P < 0.05

Category	Term	Description	Count	*P*-value
BP	GO:0006955	immune response	8	1.69E-05
BP	GO:0042330	taxis	4	1.16E-03
BP	GO:0006935	chemotaxis	4	1.16E-03
BP	GO:0050670	regulation of lymphocyte proliferation	3	5.33E-03
BP	GO:0007626	locomotory behavior	4	5.35E-03
BP	GO:0032944	regulation of mononuclear cell proliferation	3	5.46E-03
BP	GO:0070663	regulation of leukocyte proliferation	3	1.16E-03
BP	GO:0006952	defense response	5	7.77E-03
BP	GO:0009615	response to virus	3	9.04E-03
BP	GO:0008285	negative regulation of cell proliferation	4	1.14E-02

Category : GO function, Count : the number of DEGs.
Abbreviations : BP, Biological Process; DEGs, Differentially Expressed Genes; GO, Gene Ontology.

Supplementary Table 4: The KEGG pathway enrichment analysis of DEGs in the threshold of P < 0.05

Category	Term	Description	Count	*P*-value
Pathway	hsa04062	Chemokine signaling pathway	4	6.49E-03
Pathway	hsa04060	Cytokine receptor interaction	4	1.63E-02

Category : KEGG pathway, Count : the number of DEGs.
Abbreviations : DEGs, Differentially Expressed Genes; KEGG, Kyoto Encyclopedia of Genes and Genomes.
