# Supplementary material for: Epstein-Barr virus EBNA2 directs doxorubicin resistance of B cell lymphoma through CCL3 and CCL4-mediated activation of NF-κB and Btk
Source: Oncotarget. 2016 Dec 27;8(3):5361–70. doi: 10.18632/oncotarget.14243 (PMC5354914; doi:10.18632/oncotarget.14243)
Supplement: Supplementary file 2 [file oncotarget-08-5361-s002.docx]

**Supplementary Table 2: List of upregulated genes by EBNA2  in U2932 cells**

| **Gene symbol** | **Gene name** | **Fold change** | **Gene symbol** | **Gene name** | **Fold change** |
| --- | --- | --- | --- | --- | --- |
| **CCL22** | chemokine (C-C motif) ligand 22 | 10.9 | **NKX3-1** | NK3 homeobox 1 | 2.2 |
| **SNORD3A** | small nucleolar RNA, C/D box 3A | 6.8 | **NOP56** | NOP56 ribonucleoprotein homolog | 2.2 |
| **SNORD3C** | small nucleolar RNA, C/D box 3C | 6.0 | **CCL3** | chemokine (C-C motif) ligand 3 | 2.2. |
| **CCL5** | chemokine (C-C motif) ligand 5 (CCL5) | 4.3 | **CDC25A** | cell division cycle 25 homolog A | 2.2 |
| **RNU6-1** | RNA, U6 small nuclear 1 | 4.0 | **EIF4G2** | eukaryotic translation initiation factor 4 gamma, 2 | 2.2 |
| **HSPA6** | heat shock 70kDa protein 6 | 3.8 | **EIF2S1** | eukaryotic translation initiation factor 2, subunit 1 | 2.2 |
| **CCL3L3** | chemokine (C-C motif) ligand 3-like 3 | 3.5 | **RPP40** | ribonuclease P/MRP 40kDa subunit | 2.2 |
| **MET** | met proto-oncogene | 3.0. | **CARD9** | caspase recruitment domain family, member 9 | 2.2 |
| **PPP1R15A** | protein phosphatase 1, regulatory (inhibitor) subunit 15A | 2.9. | **CIRH1A** | cirrhosis, autosomal recessive 1A | 2.1 |
| **RUNX2** | runt-related transcription factor 2 | 2.7. | **NOLA1** | nucleolar protein family A, member 1 | 2.1 |
| **HSPA1A** | heat shock 70kDa protein 1A | 2.7. | **UTP14C** | UTP14, U3 small nucleolar ribonucleoprotein, homolog C | 2.1 |
| **INHBE** | inhibin, beta E | 2.6. | **SBDS** | Shwachman-Bodian-Diamond syndrome | 2.1 |
| **HSPA1B** | heat shock 70kDa protein 1B | 2.5. | **SGK1** | serum/glucocorticoid regulated kinase 1 | 2.1 |
| **UTP14A** | UTP14, U3 small nucleolar ribonucleoprotein, homolog A | 2.5 | **EWSR1** | Ewing sarcoma breakpoint region 1 | 2.1 |
| **EIF4G2** | eukaryotic translation initiation factor 4 gamma, 2 | 2.5 | **NOL6** | nucleolar protein family 6 | 2.1 |
| **EIF5** | Homo sapiens eukaryotic translation initiation factor 5 | 2.4 | **NOP56** | NOP56 ribonucleoprotein homolog | 2.1 |
| **DNAJA1** | Homo sapiens DnaJ (Hsp40) homolog, subfamily A, member 1 | 2.4 | **EIF4A1** | eukaryotic translation initiation factor 4A, isoform 1 | 2.1 |
| **NXF1** | RNA export factor 1 | 2.4 | **NFKBIZ** | nuclear factor of kappa light polypeptide gene enhancer in B-cells inhibitor, zeta | 2.1 |
| **HSPBP1** | hsp70-interacting protein | 2.3. | **CYCS** | cytochrome c, somatic | 2.1 |
| **TCEB2** | transcription elongation factor B (SIII), polypeptide 2 | 2.3. | **GAR1** | GAR1 ribonucleoprotein homolog | 2.1 |
| **POLR2L** | polymerase (RNA) II (DNA directed) polypeptide L | 2.3 | **NFKBIB** | nuclear factor of kappa light polypeptide gene enhancer in B-cells inhibitor, beta | 2.0 |
| **CD14** | CD14 molecule | 2.3 | **DUSP5** | dual specificity phosphatase 5 | 2.0 |
| **HSPA8** | heat shock 70kDa protein 8 | 2.3 | **NUP98** | nucleoporin | 2.0 |
| **CCL4L2** | chemokine (C-C motif) ligand 4-like 2 | 2.2 |  |  |  |
